# Association of JAG1 gene polymorphism with systemic blood pressure in patients with obstructive sleep apnea: a prospective cohort study

**DOI:** 10.3325/cmj.2019.60.421

**Published:** 2019-10

**Authors:** Ivana Paraničová, Viera Habalová, Lucia Klimčáková, Ivana Trojová, Jozef Židzik, Ivan Tkáč, Ružena Tkáčová, Pavol Joppa

**Affiliations:** 1 Department of Pneumology and Phtiseology, Medical Faculty of P. J. Šafárik University and L. Pasteur University Hospital, Košice, Slovakia; 2 Department of Medical Biology, Medical Faculty of P. J. Šafárik University, Košice, Slovakia; 3 Department of Internal Medicine 4, Medical Faculty of P. J. Šafárik University and L. Pasteur University Hospital, Košice, Slovakia; Paranicova et al: Association of JAG1 gene polymorphism with systemic blood pressure in patients with obstructive sleep apnea

## Abstract

**Aim:**

To assess the effects of single nucleotide polymorphisms (SNPs) on blood pressure control in patients with obstructive sleep apnea (OSA).

**Methods:**

This prospective observational cohort study, conducted between 2004 and 2014, examined the associations of SNPs of *JAG1*, *GUCY1A3-GUCY1B3*, *SH2B3*, and *NPR3-C5orf23* genes with systolic and diastolic blood pressure (SBP, DBP) in 1179 adults evaluated for OSA with overnight polysomnography. Genotyping was performed by unlabeled probe melting analysis.

**Results:**

The patients were predominantly male (69.6%, mean age 52 ± 11 years, apnea-hypopnea index 34 ± 31 episodes/h). Only *JAG1* genotype was associated with SBP and DBP: compared with AA homozygotes, G allele carriers (pooled GG and AG genotype) had significantly higher morning SBP (132 ± 19 vs 129 ± 18 mm Hg; *P* = 0.009) and morning and evening DBP (85 ± 11 vs 83 ± 10 mm Hg, *P* = 0.004; 86 ± 10 vs 84 ± 10 mm Hg, *P* = 0.012, respectively); the differences remained significant after the correction for multiple SNPs testing. In multivariate analyses, oxygen desaturation index and *JAG1* genotype independently predicted morning SBP (*P* = 0.001, *P* = 0.003, respectively) and DBP (*P* < 0.001, *P* = 0.005, respectively), and evening SBP (*P* = 0.019, *P* = 0.048, respectively) and DBP (*P* = 0.018, *P* = 0.018, respectively).

**Conclusion:**

This is the first replication study of the SNPs recently linked to arterial hypertension in general population by genome-wide association studies. Our findings suggest that *JAG1* genotype is related to blood pressure control in OSA: G allele was associated with higher morning and evening SBP and DBP.

Blood pressure is a multifactorial trait. International guidelines for the management of arterial hypertension recognize obstructive sleep apnea (OSA) as the most prevalent cause of secondary hypertension (1) and the most important factor in drug-resistant hypertension (2). Increases in OSA severity are directly linked to increases in systolic and diastolic blood pressure (SBP, DBP) and to the odds for the development of arterial hypertension (3).

Another factor that profoundly affects blood pressure control is heredity. Approximate heritability of blood pressure ranges between 30%-50% (4). The material basis for this heritability is coded in two types of genetic variants: a) major-effect genetic variants that are responsible for rare familial hypertension syndromes, contributing to blood pressure increases at the level of several millimeters of mercury (mmHg), b) small-effect genetic variants that independently influence blood pressure at the level of up to 1 mm Hg for SBP and of up to 0.5 mm Hg for DBP (4,5). Small-effect genetic variants include repetition mutations, inversions, and, most importantly, single nucleotide polymorphisms (SNPs), which may occur throughout the genome in coding, non-coding, and regulator parts of genes. Several genome-wide association studies (GWASs) revealed signals in particular genetic loci linked to blood pressure control in general population and identified over 100 SNPs associated with either blood pressure increase or arterial hypertension (6). Nevertheless, the identified loci linked to blood pressure were seldom localized inside or close to regions with known function in blood pressure control. In contrast, most of the SNPs identified by GWASs were located next to genes with pleiotropic effects, such as prenatal cardiovascular development, inter- and intracellular signaling, hematopoiesis, or metabolism of natriuretic peptides (4). Moreover, several of the newly identified SNPs linked to blood pressure control were in previous studies associated with the increased risk of coronary artery disease (7).

Although GWASs recognized the effects of certain SNPs on blood pressure regulation in the general population (5-7), there is a paucity of data about the potential effects of these SNPs in patients with pre-existing medical conditions known to modify blood pressure control. The present study aimed to evaluate the associations between blood pressure and selected gene polymorphisms with GWAS-recognized effects on blood pressure control (rs3184504 of the *SH2B3* gene, rs1327235 of the *JAG1* gene, rs13139571 of the *GUCY1A3-1B3* gene, and rs1173771 of the *NPR3c5orf23* gene) in individuals referred to a sleep laboratory for OSA evaluation. The selection of four distinct SNPs was guided by several previous studies that identified the relationships between these polymorphisms and blood pressure, arterial hypertension, and coronary artery disease in general population as summarized by Waken et al (6).

## Patients and methods

### Patients

In this prospective observational cohort study, 1179 individuals referred to the sleep unit at a tertiary referral university hospital (Department of Respiratory Medicine, L. Pasteur University Hospital, Košice, Slovakia) for a diagnostic sleep study were recruited consecutively between 2004 and 2014. They were all clinically stable adults (age over 18 years) with or without OSA. Exclusion criteria were respiratory failure, arterial hypertension secondary to a disease different from OSA, and any cardiovascular instability including accelerated hypertension. The study was approved by the L. Pasteur University Hospital Ethics Committee, and all patients gave written informed consent.

### Sleep assessment

All patients underwent overnight polysomnography (Alice 4 and Alice 5; Respironics Inc., Murrysville, PA, USA), which consisted of electroencephalography (EEG), electrooculography, electromyography, thoracic and abdominal impedance belts, thermistor flowmeter for nasal and oral airflow, microphone for snoring, and pulse oximetry. Records were scored manually following the criteria of the American Academy for Sleep Medicine (AASM) 2007 guidelines and 2012 update (8).

Apnea was defined as a reduction of airflow of ≥90% from the baseline with duration ≥10 s; hypopnea was defined as a reduction in airflow of ≥50% of baseline for ≥10 s, followed by a decrease in oxygen saturation for ≥3%, arousal, or both. Alternatively, hypopnea was identified if reduction of airflow ≥30% from the baseline was present and accompanied with a decrease in saturation ≥4%. Apnea/hypopnea index (AHI) was defined as the total number of apneic and hypopneic events per hour of sleep. Oxygen desaturation index (ODI) was calculated as the number of episodes of reductions in oxygen saturation of ≥3% per hour of sleep, and arousal index was used as a marker of sleep fragmentation characterized as the number of arousals on EEG recording per hour of sleep.

Patients with AHI less than five episodes per hour had no OSA. In patients diagnosed with OSA, its severity was classified based on AASM guidelines, with AHI≥5 and <15 episodes/h labeled as mild OSA, AHI≥15 and <30 episodes/h as moderate OSA, and AHI≥30 episodes/h as severe OSA (9).

### Blood pressure measurements

Blood pressure was measured in a sitting position by using a certified manometric device in the evening, immediately before the montage of polysomnographic leads after at least of 10 minutes of no physical effort (10), and in the morning after awakening before the removal of the leads. Systolic and diastolic blood pressure were measured three times by a trained nurse, and the mean value of the last two measurements was recorded. Manual sphygmomanometer (Welch Allyn type Gold Seriers DS66 Trigger Aneroids, Chicago, IL, USA) with annually certified accuracy of ±2.5% was used for all blood pressure measurements.

### Genotyping

Peripheral blood samples for leukocyte DNA extraction were collected by a trained nurse by venipuncture of the cubital vein in the morning after diagnostic polysomnography. DNA was extracted with the QIAamp DNA Blood Mini QIAcube Kit according to the manufacturer’s instructions on the QIAcube – robotic workstation for automated purification of DNA, RNA, or proteins (QIAGEN, Hilden, Germany). Four variants of interest were analyzed by high-resolution melting analysis (HRMA) after real-time polymerase chain reaction in the presence of LCGreen Plus dye (BioFire Defense, Salt Lake City, UT, USA) on Eco Real-Time PCR System (Illumina Inc., San Diego, CA, USA). Specifically, *SH2B3* rs3184504, *JAG1* rs1327235, *GUCY1A3* rs13139571, *NPR3* rs1173771 polymorphisms were identified by HRMA in the presence of unlabeled probe. The oligonucleotide sequences used for the analyses (Sigma-Aldrich, Haverhill, UK) are shown in Table 1. Genotypes were identified using Eco^TM^ Software 4.1. Genotyping success rate for all examined variants was 100%, and duplicate genotyping concordance was 100% (5 samples for each genotype).

**Table 1:** Oligonucleotides sequences (Sigma-Aldrich, Haverhill, UK) used in genotyping of variants of interest

Gene (risky/non-risky allele)	Oligonucleotide	Sequence 5′→3′
*SH2B3* (T/C)	forward-limit	AGCAGCTTGCTCCAGCATC
	reverse-excess	TGTAAAGGTTGTCAGGCATCTC
	probe	GAGGTCCGGCGGTGCACAC-Phos
*JAG1* (G/A)	forward-excess	CTAACCAACACTTGGCATAGACTC
	reverse-limit	CATCATGAAAATGTGAATTCAAACTCCAG
	probe	AAATCCCACGTATGCCACCAGAACAA-Phos
*GUCY1A3* (C/A)	forward-limit	ATTCCTTGTTTCCAAGTCCGGCTTC
	reverse-excess	GTGAGTATAGACAACTTCTTTCAGTTTTGC
	probe	CTACAATTTTCAATTTCTGCTCTG-Phos
*NPR3* (G/A)	forward-limit	CTGAATATTTCTGACCTTGCAGCTC
	reverse-excess	TGGGGACACAGCCAACCAT
	probe	CTGCTGGTGCTTTGTGAATAAGTTGGTAT-Phos

### Statistical analysis

Normality of the distribution was assessed by Kolmogorov-Smirnov test. The data are presented as mean ± standard deviation (SD). Differences between groups were analyzed using one way ANOVA for parametric variables and ANOVA on ranks for nonparametric variables. Holm-Sidak test was used for *post hoc* pair-wise comparisons between groups with different OSA severity. Differences in proportions between groups were analyzed with χ^2^ test. A two-tailed *P*-value of <0.05 was considered significant for comparisons between groups with different OSA severity. The alleles of the selected SNPs that confer an increase in blood pressure were referenced according to the genome database in the project Ensebml GRCh38 (11). For comparisons between the respective genetic variants for each genotyped polymorphism, the cohort was divided into two groups (a dominant model): the first group comprised the individuals with one or more copy of the risky allele (ie, conferring an increase in blood pressure) and the second group comprised the homozygotes for the non-risky allele. Consequently, after applying the Bonferroni correction for 4 tested SNPs, a *P*-value of <0.0125 was considered significant (α = 0.05/4 = 0.0125). Graphic outputs of the blood pressure comparisons between genotype groups were created with SigmaPlot version 8 (SPSS, Chicago, IL, USA) and Microsoft Powerpoint (Redmond, WA, USA). Linear relationships between blood pressure and indices of OSA severity were analyzed by calculating the Pearson correlation coefficient. In multivariate analyses, multiple linear regression models were used with blood pressure as a dependent variable, and age, sex, body mass index (BMI), antihypertensive use, ODI, and genotype as independent variables. Associations in the linear regression models are presented as nominally significant (*P* < 0.05). All analyses were carried out using SPSS, version 14 (IBM, Chicago, IL, USA).

Hardy-Weinberg equilibrium for each genotype was tested using an online calculator (*http://www.oege.org/software/hwe-mr-calc.shtml*). The genotypes were considered to be distributed as expected under Hardy-Weinberg equilibrium if the calculated χ*^2^* was <3.84 (ie, *P* value ≥0.05) (12).

## Results

### Cohort characteristics

The study involved 1179 participants (821 or 69.6% men) with mean age 52.0 ± 11.4 years (18 to 81 years; median [interquartile range] of 54.0 [45.0-60.0] years) and mean AHI 34.4 ± 30.9 episodes/h (0.1 to 157.8 episodes/h; median [interquartile range] of 24.0 [8.1-55.6] episodes/h]. A total of 202 patients had no OSA, 250 had mild OSA, 186 had moderate OSA, and 541 had severe OSA. Basic demographic data are shown in Table 2. The severity of OSA was significantly associated with male sex, older age, BMI, increasing prevalence of arterial hypertension as reported in personal history, type 2 diabetes, cardiovascular and cerebrovascular morbidity, and the use of antihypertensive drugs. All genotypes were evenly distributed among all OSA severity categories (*P* value >0.05 for each respective genotype).

**Table 2:** Demographic characteristics and polysomnographic findings in the study participants*^†^

	Entire cohort	No OSA	Mild OSA	Moderate OSA	Severe OSA	*P*
Participants (*n*)	1179	202	250	186	541	
Sex						
male	821 (70)	101 (50)	168 (67)	125 (67)	427 (79)	<0.001
female	358 (30)	101 (50)	82 (33)	61 (33)	114 (21)	
Age (years)	52.0 ± 11.4	47.2 ± 11.9	51.4 ± 11.8	52.9 ± 11.3	53.9 ± 10.4	<0.001
BMI (kg/m^2^)	32.5 ± 6.6	28.5 ± 4.8	30.2 ± 5.9	32.0 ± 5.9	35.2 ± 6.5	<0.001
Current smoker	388 (33)	67 (33)	76 (30)	53 (28)	192 (35)	0.262
Arterial hypertension	732 (62)	92 (46)	120 (48)	122 (66)	398 (74)	<0.001
Type 2 diabetes	161 (14)	7 (3)	22 (9)	29 (16)	103 (19)	<0.001
CAD	198 (17)	21 (10)	30 (12)	35 (19)	112 (21)	<0.001
MI	56 (5)	5 (2)	5 (2)	11 (6)	35 (6)	0.014
Stroke	52 (4)	4 (2)	6 (2)	9 (5)	33 (6)	0.032
Antihypertensives	680 (58)	81 (41)	116 (47)	115 (63)	368 (68)	<0.001
Alpha blockers	97 (8)	8 (4)	15 (6)	17 (9)	57 (11)	0.015
Beta blockers	333 (29)	40 (20)	49 (20)	55 (30)	189 (35)	<0.001
Ca channel blockers	291 (25)	33 (17)	47 (19)	47 (26)	164 (31)	<0.001
Diuretics	248 (21)	19 (10)	33 (13)	37 (20)	159 (30)	<0.001
ACEI	315 (27)	30 (15)	52 (21)	55 (30)	178 (33)	<0.001
Sartans	174 (15)	19 (10)	27 (11)	22 (12)	106 (20)	<0.001
NREM (min)	357.3 ± 64.3	344.3 ± 59.0	350.5 ± 59.3	347.4 ± 61.2	368.6 ± 67.7	<0.001
S1 NREM (min)	58.6 ± 49.6	49.2 ± 45.5	51.1 ± 37.2	51.9 ± 39.9	67.9 ± 57.1	<0.001
S2 NREM (min)	249.1 ± 77.8	237.9 ± 67.5	239.0 ± 65.5	233.7 ± 66.8	263.3 ± 87.4	<0.001
SWS (min)	49.5 ± 35.1	57.2 ± 35.1	60.5 ± 35.2	61.7 ± 34.7	37.4 ± 31.2	<0.001
REM (min)	60.7 ± 34.8	64.3 ± 36.0	69.0 ± 31.4	66.9 ± 34.7	53.4 ± 34.6	<0.001
AHI (episodes/h)	34.5 ± 30.9	2.4 ± 1.5	9.6 ± 2.9	21.3 ± 4.0	62.6 ± 23.4	<0.001
ODI (episodes/h)	29.5 ± 30.0	3.2 ± 8.3	7.4 ± 7.0	16.7 ± 9.7	53.8 ± 27.3	<0.001
Arousal index (episodes/h)	37.3 ± 25.2	18.0 ± 13.5	22.6 ± 12.2	28.0 ± 12.7	54.5 ± 25.0	<0.001
SpO_2_<90% (min)	58.1 ± 101.6	19.0 ± 81.0	15.0 ± 58.8	27.0 ± 73.1	103.1 ± 113.3	<0.001
Lowest SpO_2_ (%)	77.4 ± 15.8	89.4 ± 5.8	86.1 ± 5.8	81.3 ± 9.5	67.7 ± 17.1	<0.001

*OSA – obstructive sleep apnea; BMI – body mass index; CAD – coronary artery disease; MI – myocardial infarction; Ca – calcium; ACEI – inhibitors of angiotensin-converting enzyme; NREM – non-rapid eye movement sleep; S1 NREM – stage 1 NREM sleep; S2 NREM – stage 2 NREM sleep; SWS – slow wave sleep; REM – rapid eye movement sleep; AHI – apnea/hypopnea index; ODI – oxygen desaturation index; SpO_2_ – arterial oxygen saturation measured by pulse oximetry.

†Data are presented as number (n) and percentage (%) or mean ± standard deviation, unless otherwise stated. *P*-values were calculated using one way ANOVA and χ^2^ test as appropriate.

### Association of OSA with blood pressure

Morning and evening SBP and DBP increased with increasing OSA severity, with the highest values in patients with severe OSA (Table 3). Morning SBP and DBP were positively correlated with AHI (*R* = 0.212 and *R* = 0.210, respectively), arousal index (*R* = 0.200 and *R* = 0.191, respectively), desaturation index (*R* = 0.239 and *R* = 0.216, respectively), and time spent in saturation under 90% (*R* = 0.182 and *R* = 0.158, respectively), with *P* < 0.001 for all. Evening SBP and DBP were positively correlated with AHI (*R* = 0.171 and *R* = 0.129, respectively), arousal index (*R* = 0.138 and *R* = 0.107, respectively), and desaturation index (*R* = 0.186 and *R* = 0.145, respectively), with *P* < 0.001 for all, and evening SBP was correlated with time in desaturation under 90% (*R* = 0.094, *P* = 0.002).

**Table 3:** Blood pressure values in participants grouped by severity of obstructive sleep apnea (OSA)*

	No OSA	Mild OSA	Moderate OSA	Severe OSA	*P*
**Participants (n)**	202	250	186	541	
Morning systolic blood pressure (mmHg)	126.7 ± 19.1	126.4 ± 16.7	130.2 ± 17.3	135.5 ± 18.5^†‡§^	<0.001
Morning diastolic blood pressure (mmHg)	81.7 ± 11.5	82.7 ± 10.3	83.6 ± 10.0	87.1 ± 10.9^†‡§^	<0.001
Evening systolic blood pressure (mmHg)	128.7 ± 16.3	131.8 ± 16.3	132.5 ± 18.1	136.5 ± 18.3^†‡^	<0.001
Evening diastolic blood pressure (mmHg)	83.3 ± 9.2	84.9 ± 8.8	85.0 ± 10.3	86.6 ± 10.2^†^	0.003

*Data are presented as mean ± standard deviation, unless otherwise stated. *P*- values were calculated using one way ANOVA.

^†^*P*-value <0.05 vs no OSA.

^‡^*P*-value <0.05 vs mild OSA.

^§^*P*-value <0.05 vs moderate OSA.

### Association of selected genotypes with blood pressure

Significant differences in SBP and DBP were observed between the genotype groups of polymorphism in the *JAG1* gene. Alleles A and G of *JAG1* gene polymorphism were distributed according to Hardy-Weinberg equilibrium. Sex distribution, age, BMI, AHI, ODI, antihypertensive medication use, and the proportion of patients according to OSA severity were similar in the two *JAG1* genotype groups (Table 4).

**Table 4:** Basic demographic characteristics in participants grouped by *JAG1* genotype*^†^

	*JAG1* polymorphism genotype		*P*
	AA	AG+GG	
Participants (*n*)	360	819	
Male sex	252 (70)	569 (69)	0.911
Age (years)	52.5 ± 10.6	51.8 ± 11.7	0.420
BMI (kg/m^2^)	32.4 ± 6.7	32.5 ± 6.5	0.581
Antihypertensive medication users	207 (58)	473 (58)	0.965
AHI (episodes/h)	34.2 ± 31.6	34.6 ± 30.6	0.719
ODI (episodes/h)	29.2 ± 31.2	29.7 ± 29.4	0.424
No OSA	61 (17)	141 (17)	0.358
Mild OSA	82 (23)	168 (21)	
Moderate OSA	64 (18)	122 (15)	
Severe OSA	153 (42)	388 (47)	

*BMI *–* body mass index; AHI *–* apnea-hypopnea index; ODI *–* oxygen desaturation index; OSA *–* obstructive sleep apnea.

†Data are presented as number (n) and percentage (%) or mean ± standard deviation, unless otherwise stated.

**Figure 1:**
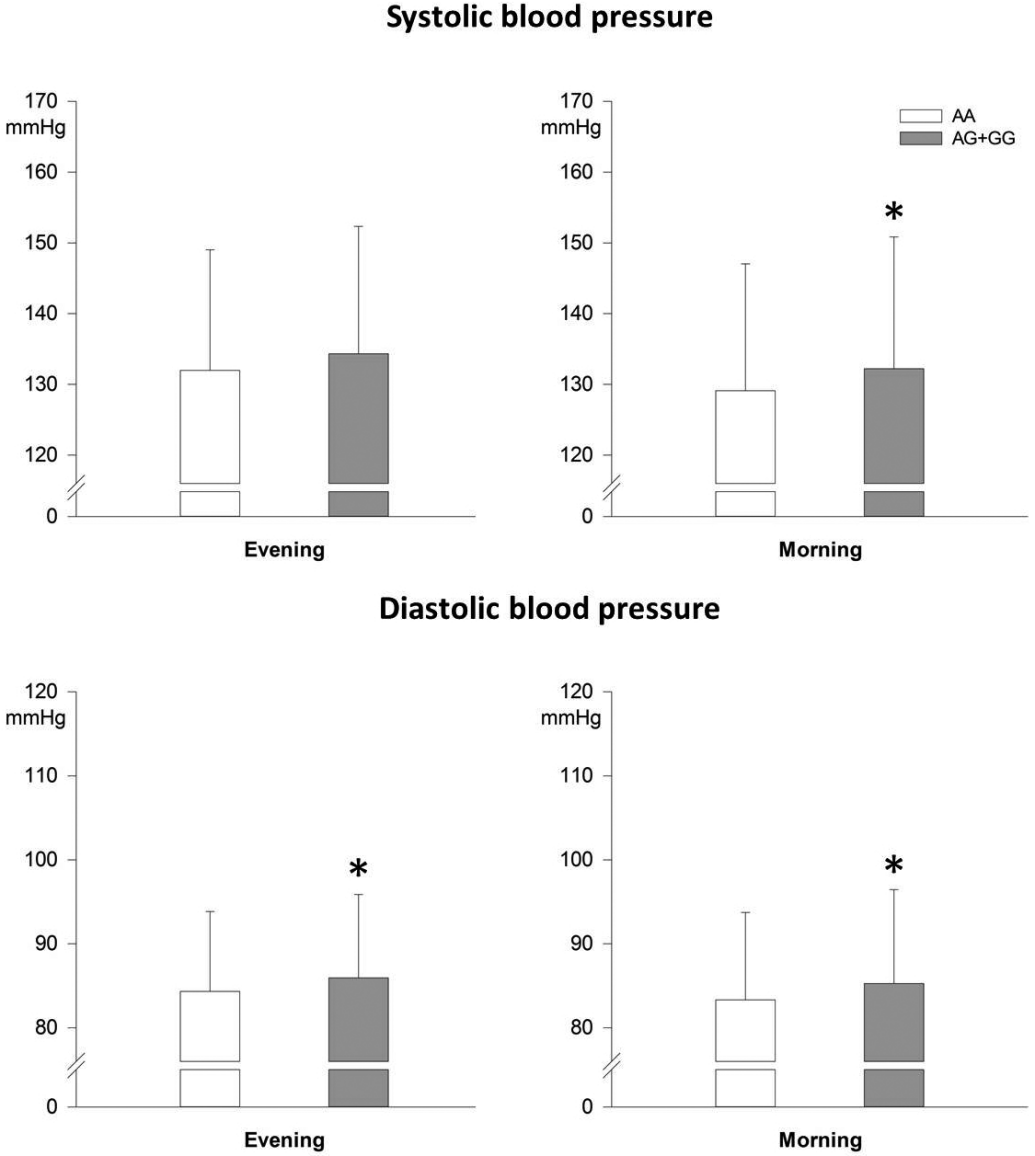
Comparison of the systolic and diastolic blood pressure between AA homozygotes and G allele carriers of *JAG1* gene polymorphism; *P-value <0.0125 vs AA homozygotes. White – AA; gray – AG+GG.

*JAG1* polymorphism genotypes were associated with blood pressure in the entire cohort (Figure 1). After applying the Bonferroni correction for 4 genotyped SNPs, allele G carriers (pooled homozygotes GG and heterozygotes AG) had significantly higher morning SBP (132.2 ± 18.6 vs 129.1 ± 17.9 mm Hg, *P* = 0.009), morning DBP (85.3 ± 11.2 vs 83.3 ± 10.4 mm Hg, *P* = 0.004), and evening DBP (85.9 ± 9.9 vs 84.3 ± 9.5 mm Hg, *P* = 0.012) than allele A homozygotes.

We also examined the association of OSA severity with blood pressure in the distinct *JAG1* polymorphism genotype groups. G allele carriers with severe OSA had significantly higher morning and evening SBP and DBP (*P* < 0.05 for all) than G allele carriers with no OSA. In addition, AA homozygotes with severe OSA had higher morning SBP and DBP (*P* < 0.05 for both), and evening SBP (*P* < 0.05) compared with AA homozygotes with no OSA (Table 5).

**Table 5:** Blood pressure across obstructive sleep apnea severity groups in AA homozygotes and G allele carriers of the *JAG1* gene polymorphism*^†^

	No OSA	Mild OSA	Moderate OSA	Severe OSA	*P*
Morning SBP					
AA	123.4 ± 13.8	123.9 ± 16.5	127.8 ± 17.5	134.7 ± 18.8^‡§^	<0.001
AG+GG	128.1 ± 20.9	127.6 ± 16.7	131.5 ± 17.1	135.9 ± 18.3^‡§^	<0.001
Morning DBP					
AA	79.2 ± 8.9	81.5 ± 9.6	82.1 ± 9.6	86.5 ± 10.8^‡§^	<0.001
AG+GG	82.7 ± 12.3	83.2 ± 10.6	84.3 ± 10.1	87.4 ± 11.0^‡§^	<0.001
Evening SBP					
AA	125.5 ± 12.7	130.3 ± 15.6	132.2 ± 20.2	135.2 ± 17.3^‡^	0.003
AG+GG	130.1 ± 17.5	132.6 ± 16.7	132.6 ± 16.9	137.0 ± 18.7^‡^	0.001
Evening DBP					
AA	82.6 ± 8.7	84.1 ± 8.5	84.5 ± 10.6	85.0 ± 9.9	0.438
AG+GG	83.6 ± 9.4	85.3 ± 8.9	85.3 ± 10.2	87.2 ± 10.3^‡^	0.010

*OSA – obstructive sleep apnea; SBP – systolic blood pressure; DBP – diastolic blood pressure.

†Data are presented as mean ± standard deviation in millimeters of mercury (mmHg). *P-*values were calculated using one way ANOVA.

^‡^*P*-value <0.05 vs no OSA.

^§^*P*-value <0.05 vs mild OSA.

To further investigate the association of both intermittent hypoxia and *JAG1* genotype with blood pressure control, we performed multivariate analyses using multiple linear regression models with each respective blood pressure value as a dependent variable and age, sex, BMI, use of antihypertensive medication, ODI, and *JAG1* genotype as independent variables. Both ODI and *JAG1* genotype were significant and independent predictors of morning and evening SBP and DBP (*P* < 0.05 for both, Table 6). Introducing AHI instead of ODI as an index of OSA severity in multivariate linear models yielded similar results (data not shown). Finally, we also analyzed a potential independent association of an interaction term *JAG1* genotype*ODI with blood pressure by adding the interaction term to the previous models. Nevertheless, the *JAG1* genotype*ODI interaction term was not a significant predictor of either SBP or DBP in any of the models.

**Table 6:** Multivariate models with systolic and diastolic blood pressure as dependent variables*

	Systolic blood pressure						Diastolic blood pressure					
	morning			evening			morning			evening		
	β	standard error	*P*	β	standard error	*P*	β	standard error	*P*	β	standard error	*P*
Age	0.348	0.050	<0.001	0.191	0.051	<0.001	0.089	0.031	0.004	0.032	0.029	0.277
Sex	-0.458	1.122	0.683	-0.757	1.156	0.513	-1.510	0.697	0.031	-1.726	0.664	0.009
Body mass index	0.348	0.098	<0.001	0.238	0.100	0.017	0.152	0.061	0.013	0.072	0.057	0.208
Antihypertensive use	4.671	1.200	<0.001	5.031	1.223	<0.001	1.895	0.746	0.011	1.080	0.702	0.124
Oxygen desaturation index	0.068	0.021	0.001	0.051	0.021	0.019	0.045	0.013	<0.001	0.029	0.012	0.018
*JAG1* genotype	3.211	1.085	0.003	2.201	1.113	0.048	1.884	0.674	0.005	1.512	0.639	0.018

*P-value for each variable in the model presented. Model for morning systolic blood pressure R^2^ = 0.16; F = 37.5, *P* < 0.001; model for evening systolic blood pressure R^2^ = 0.10; F = 19.0, *P* < 0.001; model for morning diastolic blood pressure R^2^ = 0.08, F = 17.6, *P* < 0.001; model for evening diastolic blood pressure R^2^ = 0.04, F = 7.1, *P* < 0.001.

No significant associations between the further three tested SNPs (*SH2B3*, *GUCY1A3-GUCY1B3*, and *NPR3c5orf23*) and blood pressure were observed (Table 7).

**Table 7:** Associations between single-nucleotide polymorphisms *SH2B3*, *GUCY1A3-GUCY1B3*, and *NPR3c5orf23* and blood pressure*^†^

Single-nucleotide polymorphism	Risky/non-risky allele (participant)	*P*			
		morning SBP	morning DBP	evening SBP	evening DBP
*SH2B3*	CC (268)/CT+TT (911)	0.414	0.734	0.801	0.207
*GUCY1A-GUCY1B*	AA (93)/AC+CC (1086)	0.322	0.172	0.679	0.292
*NPR3C5orf23*	AA (199)/AG+GG (980)	0.362	0.791	0.088	0.076

*SBP – systolic blood pressure; DBP – diastolic blood pressure.

†*P*-value for comparisons of the respective blood pressures between the two genotype groups for each single-nucleotide polymorphism is presented.

## Discussion

The present genetic association study revealed that among patients with suspected OSA, carriers of the risky G allele of the rs1327235 polymorphism of *JAG1* gene had significantly higher morning SBP and DBP, and evening DBP than AA homozygotes. The association of *JAG1* genotype with blood pressure was independent of age, sex, BMI, and use of antihypertensive medication. Moreover, our data indicate that OSA severity has deleterious effects on blood pressure independently of the effect carried by the *JAG1* genotype. Previously, several GWASs reported links between *JAG1* genotype and increased SBP and DBP, and higher odds of hypertension and coronary artery disease in the general population (5,13). In individuals of European descent, effect size estimate of coded allele G was 0.340 mm Hg for SBP, 0.302 mm Hg for DBP, and 0.034 in odds for hypertension (5). Furthermore, Wain et al (13) reported a relationship between rs1327235 and the mean arterial pressure and pulse pressure in healthy individuals. Our study is the first to replicate the GWAS-documented association between the rs1327235 polymorphism of the *JAG1* gene and blood pressure control, and to suggest that the rs1327235 polymorphism of *JAG1* gene may be involved in blood pressure regulation in patients with OSA.

The prevalence of hypertension in OSA ranges from 30 to 85%, while the prevalence of OSA among hypertensive patients is 40% (14). The association between OSA and hypertension is independent of confounders such as obesity, smoking, or alcohol intake (15). Increasing severity of sleep apnea is proportional to the increase in blood pressure and incidence of hypertension (15). Moreover, OSA has been identified as a prevalent underlying condition in drug-resistant hypertension (2,16). An important mediator for systemic hypertension in patients with OSA is chronic intermittent hypoxia (15). This type of hypoxia contributes to additional factors related to increases in blood pressure: sympathetic activation, blunted baroreflex sensitivity (17), increased chemosensory activity of the carotid body (18), and endothelial dysfunction (19). In the studied cohort, the differences in blood pressures between the *JAG1* genotype groups were 2.0-4.5 mm Hg for SBP and 0.9-3.5 mm Hg for DPB across different OSA severity groups. Interestingly, in a GWAS, the mean estimated effect size of singular polymorphism was 0.5 mm Hg for DBP and 1.0 mm Hg for SBP (5). Therefore, the differences observed in our study exceeded the average of the expected effect size for a singular polymorphism. On the other hand, differences in blood pressures attributed to a singular gene polymorphism in our and other studies (5) are lower than the reported effect size of mutations in familial hypertensive syndromes, which was 10 mm Hg for SBP (4). In the present study, the differences in SBP and DBP between patients with severe OSA and those without OSA were up to 9 and 6 mm Hg, respectively. These results are comparable to those observed by Peppard et al (15) in their landmark prospective study.

Several genetic association studies have reported links between genetic background and arterial hypertension in OSA. Lavie et al (20) reported that OSA patients with haptoglobin phenotype 2-2 had increased susceptibility to hypertension compared with OSA patients with haptoglobin phenotype 2-1. Riha et al (21) demonstrated an association between tumor necrosis factor-alpha polymorphism and OSA severity; nevertheless, no relationship between tumor necrosis factor-alpha polymorphism and hypertension was observed. Considering the crucial role of sympathetic nervous system activation in the OSA-related arterial hypertension, genetic background of β_2_-adrenergic receptor was also investigated: heterozygosity for the β_2_-adrenergic receptor was associated with a lower rate of post-myocardial infarction survival in patients with OSA and a high cardiovascular risk profile. However, functionally relevant polymorphisms of β2-adrenergic receptor did not modify blood pressure induced by OSA (22). In another study, β_1_-adrenergic receptor gene Arg389Arg genotype was associated with an increased prevalence of hypertension in individuals with mild OSA (23). The results on the role of angiotensin-converting enzyme gene insertion/deletion (I/D) polymorphism in blood pressure in patients with OSA yielded conflicting results. While in one study, the D allele of the I/D polymorphism was linked with central obesity but not with OSA or hypertension (24), in another it was associated with hypertension only (25). Interestingly, Patel et al (26) observed a protective effect of D allele against hypertension in severe OSA, and Boström (27) reported a significant interaction between OSA and I/D polymorphism and the prevalence of hypertension in OSA.

Our study was the first to analyze, in patients with OSA, the potential role of polymorphisms originally identified in GWASs as being significant in blood pressure regulation in general population. Therefore, our results on the associations between the *JAG1* gene polymorphism and morning and evening SBP and DBP in patients with OSA confirm and extend the previous results on the role of *JAG1* genotype in blood pressure control. *JAG1* gene is localized on chromosome 20. It codes jagged 1 protein that serves as a ligand for multiple Notch receptors. Notch signaling is an evolutionary conserved short-range intercellular pathway with a role in many essential processes, such as cell fate determination during embryonic development, vascular morphogenesis, angiogenesis, phenotypic switching, and vascular remodeling after injury (28). Recently, *JAG1* has been implicated in the pathogenesis of pulmonary arterial hypertension by aggravating pulmonary vascular remodeling both in animal studies (29) and in patients with tetralogy of Fallot and pulmonary stenosis (30). Mechanisms underlying the association between *JAG1* genotype and systemic hypertension might parallel those observed in pulmonary hypertension, but experimental data for this hypothesis are lacking. Of note, a human clinical condition with autosomal dominant heritability, termed Alagille syndrome, involves both major frameshift mutations of *JAG1* gene and systemic arterial hypertension (31). Nevertheless, direct pathophysiological links between *JAG1* genotype and blood pressure control remain to be elucidated.

The main strength of our study is a distinct cohort of patients who underwent full attended polysomnography, which is a fundamental method in diagnosing sleep disordered breathing. Nevertheless, several limitations to the current study need to be also acknowledged. First, only a minority of the genetic variants significantly associated with SBP, DBP, and hypertension in GWAS was near a gene with a recognized role in blood pressure control (4). Moreover, the effect size of each individual variant is very small, and even collectively, the 29 variants explain only 1%-2% of SBP and DBP variance (5). This may clarify the lack of associations between blood pressure and three of the four SNPs tested in the present investigation; further studies in much larger cohorts are needed to analyze such potential associations in more detail. Second, although our results suggest a role for the *JAG1* gene in blood pressure control in patients with OSA, investigation of the underlying mechanisms of the observed association was beyond the scope of the present study. Therefore, the present results should be considered hypothesis-generating, and further studies are needed to analyze the relationships between the *JAG1* gene polymorphism and blood pressure control in OSA in more detail.

In conclusion, the present study suggests that the risky G allele in the *JAG1* genotype is associated with higher morning and evening SBP and DBP in patients referred to sleep laboratory for the evaluation of suspected OSA. Large-scale genetic studies are needed to elucidate the impact of susceptibility polymorphisms that contribute to hypertension in such patients.
